# Supplementary material for: Estimation of the Actual Incidence of Coronavirus Disease (COVID-19) in Emergent Hotspots: The Example of Hokkaido, Japan during February–March 2020
Source: J Clin Med. 2021 May 28;10(11):2392. doi: 10.3390/jcm10112392 (PMC8198150; doi:10.3390/jcm10112392)
Supplement: Supplementary file 1 [file jcm-10-02392-s001.zip › jcm-1165797-supplementary.pdf]

Journal of
*Clinical Medicine*

MDPI

# Supplementary Materials

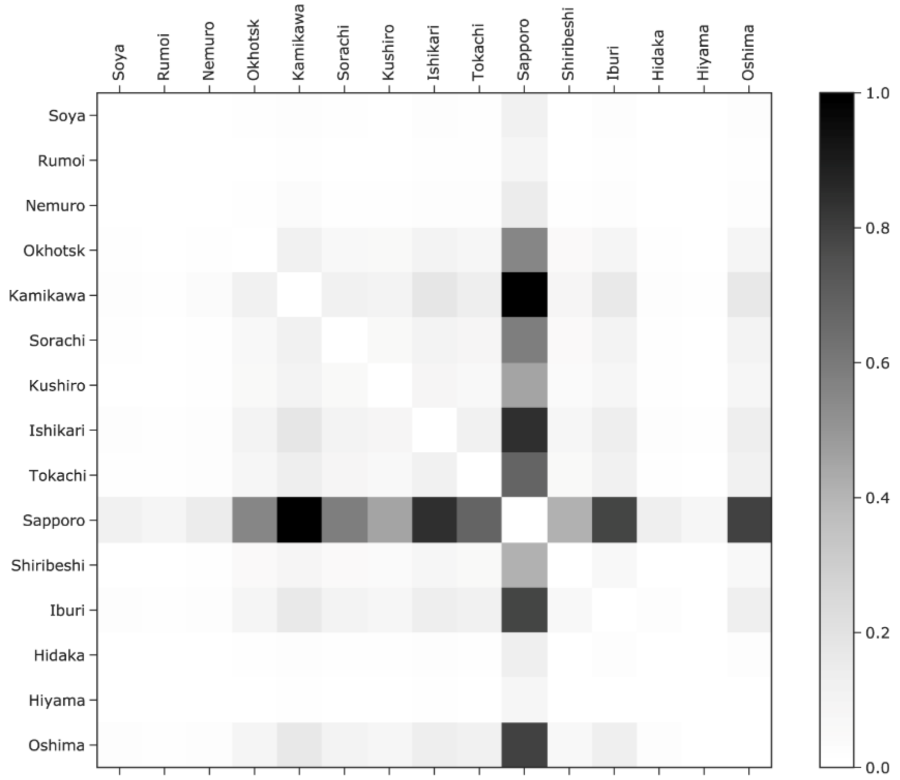


**Figure S1.** Reconstructed connectivity matrix between subprefectures of Hokkaido. Ishikari subprefecture was separated into two subregions: within Sapporo and outside of Sapporo. The grey-shaded heatmap indicates the relative intensity of travel volume. The resulting connectivity matrix was forced to be symmetric.

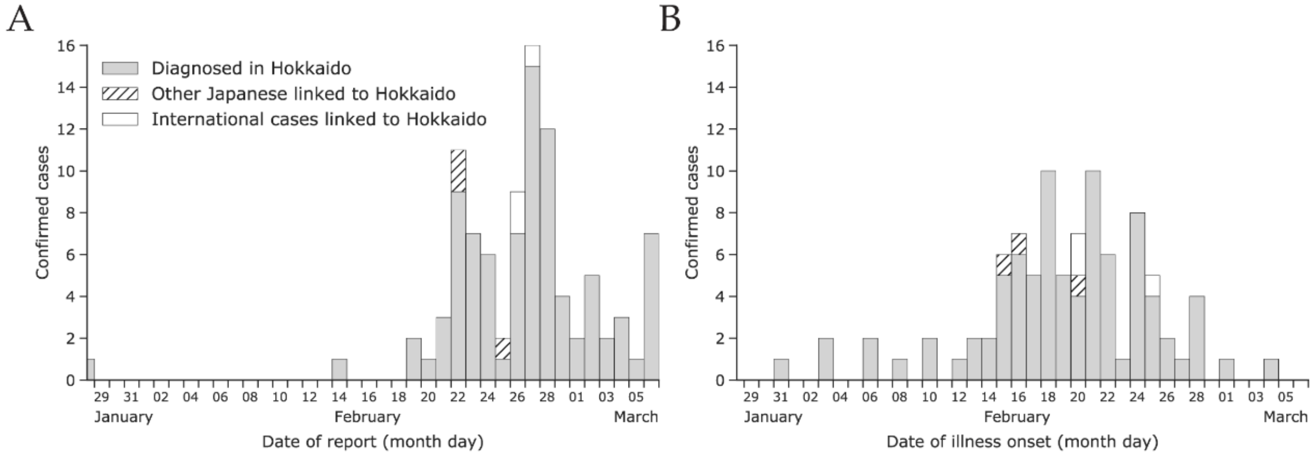


**Figure S2.** Epidemic curves by date of confirmation (**A**) and date of illness onset (**B**) as of 6 March 2020 for confirmed cases among Japanese nationals linked to Hokkaido.
